# Zebrafish studies identify serotonin receptors mediating antiepileptic activity in Dravet syndrome

**DOI:** 10.1093/braincomms/fcz008

**Published:** 2019-08-01

**Authors:** Aliesha L Griffin, Priyadarshini Jaishankar, Jean-Marc Grandjean, Steven H Olson, Adam R Renslo, Scott C Baraban

**Affiliations:** 1 Epilepsy Research Laboratory and Weill Institute for Neuroscience, Department of Neurological Surgery, University of California San Francisco, San Francisco, CA 94122, USA; 2 Department of Pharmaceutical Chemistry and Small Molecule Discovery Center, University of California San Francisco, CA 94143, USA; 3 Department of Neurology, Institute for Neurodegenerative Diseases and Weill Institute for Neurosciences, University of California San Francisco, San Francisco, CA 94143, USA

**Keywords:** Chemical biology, epilepsy, serotonin, electrophysiology, drug development

## Abstract

Dravet syndrome is a life-threatening early-onset epilepsy not well controlled by antiepileptic drugs. Drugs that modulate serotonin (5-HT) signalling, including clemizole, locaserin, trazodone and fenfluramine, have recently emerged as potential treatment options for Dravet syndrome. To investigate the serotonin receptors that could moderate this antiepileptic activity, we designed and synthesized 28 novel analogues of clemizole, obtained receptor binding affinity profiles, and performed *in vivo* screening in a *scn1lab* mutant zebrafish (*Danio rerio*) model which recapitulates critical clinical features of Dravet syndrome. We discovered three clemizole analogues with 5-HT receptor binding that exert powerful antiepileptic activity. Based on structure–activity relationships and medicinal chemistry-based analysis, we then screened an additional set of known 5-HT receptor specific drug candidates. Integrating our *in vitro* and *in vivo* data implicates 5-HT_2B_ receptors as a critical mediator in the mechanism of seizure suppression observed in Dravet syndrome patients treated with 5-HT modulating drugs.

## Teaser

Serotonin drugs, including clemizole, have recently emerged as potential treatment options for Dravet syndrome. We synthesized a library of clemizole analogues and screened for anti-seizure activity using the zebrafish Dravet syndrome model. Coupled with *in vitro* binding, we identified 5-HT_2B_ receptors as a mediator in the mechanism of seizure suppression.

## Graphical Abstract

**Graphical Abstract:**
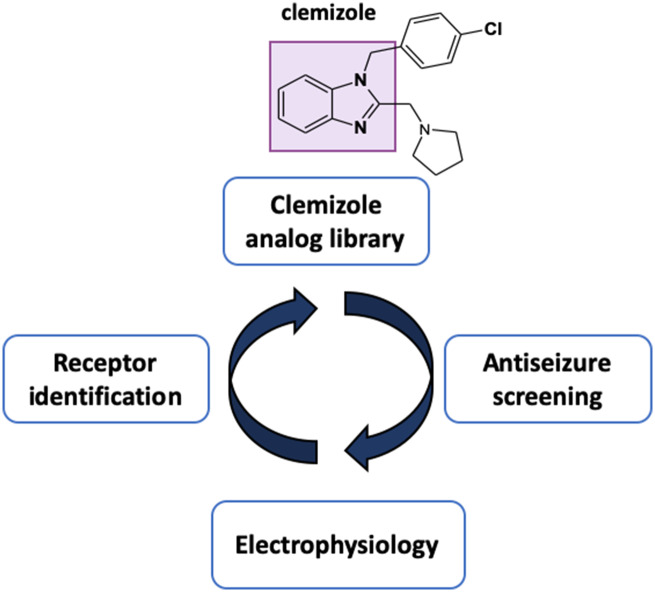

## Introduction

The discovery of antiepileptic drugs (AEDs) has traditionally relied upon acute seizure models in adult rodents. Unfortunately, this approach does not incorporate genetic epilepsies like those associated with *de novo* gene mutations and classified as catastrophic childhood epileptic encephalopathies (Löscher, 2017*a*,*b*). *In vivo* drug discovery and development using phenotype-based screening in genetically modified larval zebrafish (*Danio rerio*) offer a promising ‘precision medicine’ focused alternative (Baraban *et al.*, 2013; Dinday and Baraban, 2015; Griffin *et al.*, 2016; Grone *et al.*, 2017). Drug candidates identified using *in vivo* screens provide valuable biological context and offer a more successful route to clinical implementation. As an example, about 62% of first-in-class small molecules registered by the Food and Drug Administration between 1999 and 2008 had their origin in a phenotype-based approach compared to only 38% in target-based drug discovery (Swinney and Anthony, 2011). Zebrafish are uniquely suited to phenotype-based *in vivo* drug discovery programs and are highly amenable to the study of structure–activity relationships that represent a critical step in the drug development process (Zon and Peterson, 2005).

Dravet syndrome is a medically refractory catastrophic epileptic encephalopathy. Patients typically exhibit frequent episodes of prolonged seizures before the age of one (Dravet, 2011), and have an increased risk of Sudden Unexplained Death in Epilepsy (Cooper *et al.*, 2016). The majority of Dravet syndrome patients are identified with *de novo* mutations within the *SCN1A* gene resulting in haploinsufficiency of a neuronal voltage-gated sodium channel (Catterall *et al.*, 2010; Escayg and Goldin, 2010; Dravet and Oguni, 2013). Current Food and Drug Administration-approved antiepileptic drugs are unable to provide adequate seizure control in this patient population. The lack of effective seizure control using available antiepileptic drugs has resulted in a significant effort to discover and develop new treatment options for these patients (Ziobro *et al.*, 2018). Although genetically modified mouse models and induced pluripotent stem cell derived neurons exist to model features of Dravet syndrome, large-scale drug discovery and development in these systems is lacking. As an alternative, larval zebrafish with a mutation in the *SCN1A* homologue, *scn1lab*, display persistent spontaneous electrographic seizures and convulsive swim behaviours, as early as 3 days post-fertilization (Baraban *et al.*, 2013). Importantly, these spontaneous seizures in *scn1lab* mutant zebrafish exhibit pharmaco-resistance to 13 antiepileptic drugs (Baraban *et al.*, 2013; Ziobro *et al.*, 2018). As such, this zebrafish model faithfully mimics the epileptic phenotype observed in Dravet syndrome patients. Using *scn1lab* zebrafish and a two-stage *in vivo* phenotype-based platform, blind screening of more than 3500 compounds identified serotonin (5-HT) receptor agonists (clemizole, trazodone and lorcaserin) and a serotonin reuptake inhibitor (fenfluramine) as potential new Dravet syndrome therapies (Baraban *et al.*, 2013; Dinday and Baraban, 2015; Sourbron *et al.*, 2016; Griffin *et al.*, 2017). Furthermore, limited clinical studies of lorcaserin (Griffin *et al.*, 2017; Tolete *et al.*, 2018), trazodone (Kauppila *et al.*, 2018), and fenfluramine (Ceulemans *et al.*, 2012; Ceulemans *et al.*, 2016; Schoonjans *et al.*, 2017) have demonstrated anti-seizure activity in Dravet syndrome patients.

Serotonin receptors (5-HTR) represent a group of G-protein coupled receptors and ligand-gated ion channels found in the central and peripheral nervous system (Barnes and Sharp, 1999). 5-HTRs can be divided into seven subtypes, with subunits 5-HT_1__–__7_ receptors expressed in neurons of the central nervous system, and 5-HT_1A_R_,_ 5-H_2A_R and 5-H_2B_R also present on astrocytes (Carson *et al.*, 1996; Zhang *et al.*, 2010; Miyazaki and Asanuma, 2016). Since the 1970s, there has been a growing body of evidence to implicate 5-HT with seizure susceptibility; however, the precise mechanism remains elusive. Increased brain 5-HT was reported to reduce the severity of audiogenic- and chemoconvulsant-induced seizures (De la Torre *et al.*, 1970; De la Torre and Mullan, 1970), as well as increase the threshold to electroshock-induced seizures (Kilian and Frey, 1973). More recently, 5-HT_2C_R knockout mice show spontaneous seizures, premature death (Tecott *et al.*, 1995) and are more sensitive to electroshock- or chemoconvulsant-induced seizure protocols (Applegate and Tecott, 1998). Furthermore, studies in DBA/2 mice support a role for 5-HTR modulation in Sudden Unexplained Death in Epilepsy (Tupal and Faingold, 2006; Uteshev *et al.*, 2010).

Here, we report a structure–activity relationship study designed to test the hypothesis that 5-HTRs are a critical site of action for drugs showing anti-seizure activity in Dravet syndrome patients. We synthesized 28 novel clemizole analogues (clemalogues) with varying 5-HT_2_R binding affinities and identified three clemalogues exerting a powerful suppression of convulsive swim behaviour and electrographic seizure activity in *scn1lab* zebrafish. Based on these 5-HT_2_R properties, a medicinal chemistry structure–activity relationship-based analysis led to a second round of screening, and identification of two 5-HT_2B_R agonists (methylergonovine and 6-(2-aminopropyl)benzofuran (6-APB)) that also suppressed convulsive swim behaviour and electrographic seizure activity. Taken together, these findings have implications for the mechanism of action of 5-HT_2_R modulating drugs (i.e. clemizole, lorcaserin, trazodone and fenfluramine) currently in clinical development for Dravet syndrome (Knupp and Wirrell, 2018; Ziobro *et al.*, 2018) and for future research into how 5-HT signalling may influence seizure activity.

## Materials and methods

### Chemical synthesis and compounds

Initially, clemizole analogues were synthesized in house at the UCSF Small Molecule Discovery Center for functional testing. Select compounds were also synthesized independently by Oxygen Healthcare Research Pvt. Ltd. for confirmation purposes using the same UCSF methodology outlined. Chemical reagents and solvents used here are commercially available, unless stated otherwise. For all air and/or moisture sensitive reactions experiments were performed under an argon atmosphere using oven-dried glassware and commercially available anhydrous solvents. All reagents that were deemed air and/or moisture sensitive were transferred via syringe or cannula through rubber septa into the reaction vessel. A rotary evaporator, set to ca. 10–50 Torr, was used for solvent removal. A Varian INOVA-400 400 MHz spectrometer was used to measure 1H nuclear magnetic resonance (NMR) spectra and reported in δ units (ppm). NMR spectra were referenced relative to residual NMR solvent peaks and coupling constants (*J*) are reported in hertz (Hz). A CEM Discover microwave reactor was used to carry out microwave reactions. An Isolera Four flash chromatography system and SiliaSep silica gel cartridges (Silicycle) were used for column chromatography. A Waters Micromass ZQ mass spectrometer equipped with Waters 2795 Separation Module, Waters 2424 Evaporative Light Scattering Detector, and Waters 2996 Photodiode Array Detector was used to acquire liquid chromatography/mass spectrometry data. Separations were performed on a XTerra^®^ MS C18, 5 µm, 4.6 × 50 mm column, at ambient (unregulated) temperature using a mobile phase of water–methanol containing 0.1% formic acid. Details of synthesis steps for each distinct synthetic route employed are described in the Supplementary material.

Compounds were commercially sourced from Millipore Sigma [Methylergonovine maleate, BW-723C86, 1-(3-chlorophenyl)piperazine hydrochloride (m-CPP), (+)-Norfenfluramine hydrochloride], Cayman Chemicals [Cabergoline, Bromocriptine mesylate, (−)-Apomorphine hydrochloride], ApexBio (Ro 60-0175 fumarate), Tocris Bioscience (CP-809, 101 hydrochloride), AK Scientific, Inc. (Piribedil) and Axon Medchem (TL 99 hydrobromide). Ten millimolars of compound stock solutions were made in dimethyl sulfoxide and then diluted in embryo medium for assays.

### Zebrafish maintenance

Zebrafish (*D. rerio*) was maintained in our zebrafish facility on a 14/10 h light dark cycle. Experimental procedures followed the Guide for the Care and Use of Animals (ebrary Inc. 2011) and were approved by the Institutional Animal Care and Use Committee (protocol # AN108659-03). Embryos were obtained by natural spawning of adult heterozygous *scn1lab* (didy^s552^) animals maintained on a Tubingen long fin strain background. Starting at 3 days post-fertilization homozygous *scn1lab* mutants (*n* = 2500) appear visibly darker than age-matched wild-type larvae.

### Seizure monitoring

For locomotion studies, zebrafish larvae (5 days post-fertilization) were placed into a single well of a clear flat-bottomed 96-well microplate containing embryo media. Sex determination is not possible at this stage of development. All drug screening experiments were conducted in an unbiased manner by investigators blinded to the test compounds and all files coded for *post hoc* analysis. The 96-well plate containing larvae was then placed inside a DanioVision box where they were allowed to acclimate (20 min; room temperature). EthoVision XT software (DanioVision, Noldus Information Technology) was used to obtain locomotion plots (10 min in duration). Seizure scoring was performed, as described (Baraban *et al.*, 2005). All locomotion plots were analysed for distance travelled (in millimetres) and mean velocity (in millimetres per second). After 90 min of drug exposure larvae were examined for toxic side-effects. Compounds that decreased or stopped the larva heartbeat, or reduced or eliminated the escape response when touched, were considered toxic.

For electrophysiology studies, zebrafish larvae were anaesthetized with cold by placing at 4°C for 5 min until no movement was observed and then immobilized in 1.2% agarose dorsal side up. Using a glass micro-electrode positioned under an upright microscope, local field potential (LFP) recordings were obtained from forebrain or optic tectum structures, as described (Baraban *et al.*, 2005). Agarose-embedded LFP recording sessions (10 min in duration) were obtained using Axoclamp software (Molecular Devices; Sunnyvale, CA, USA) and sampled at 1 kHz. Epileptiform events were identified *post hoc*. These were classified as multi-spike or poly-spike upward or downward membrane deflections greater than 3× baseline noise level and 150–250 ms in duration (interictal-like) or greater than 5× baseline noise, multi-spike and >500 ms in duration (ictal-like); both events were counted using threshold detection settings in Clampfit (Molecular Devices; Sunnyvale, CA, USA). Agarose-embedded larvae were continuously monitored for blood flow and heart rate using an Axiocam digital camera at video frame rate.

### Receptor binding assays

All *in vitro* binding assay and Ki data studies were performed by the US National Institute of Mental Health Psychoactive Drug Screening Program (NIMH PDSP). For these studies, drugs were screened against recombinant, stably expressed human 5-HT_2A_R, 5-HT_2B_R, 5-HT_2C_R and H1, as described at https://pdspdb.unc.edu/pdspWeb/ (Besnard *et al.*, 2012).

### Statistical analysis

For behaviour analysis, the threshold for a change in mean swim velocity ≥40% is considered significant (>1.5× SD of 250 control treated *scn1lab*). Unless otherwise indicated, all data in this manuscript are presented as the mean ± standard error of the mean. For comparison between more than two groups a one-way analysis of variance test was performed. n the A non-parametric Kruskal–Wallis test was used followed by Dunns multiple comparison test for variance data that did not exhibit a normal distribution. Statistically significant differences are indicated with asterisks (*P <* *0.05; **P <* *0.01; ***P <* *0.001).

### Data availability statement

The authors confirm that the data supporting the findings of this study are available within the article and Supplementary material.

## Results

### Design, synthesis and whole-organism screening of clemizole analogues

**Figure 1:**
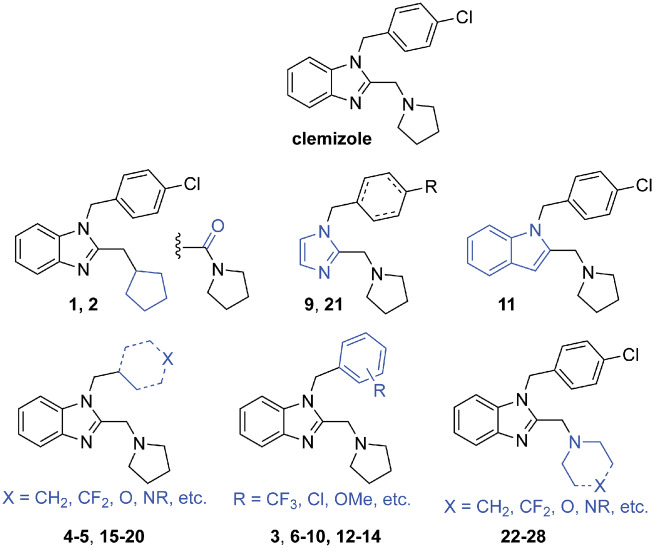
**Structure of clemizole and clemalogues 1–28.** Chemical structure of clemizole and 28 clemizole analogues synthesized as part of SAR studies detailed herein. Sites of modification to the clemizole structure are highlighted in blue for each clemalogue subtype shown. Full chemical structures are provided in Supplementary Table S1.

**Figure 2:**
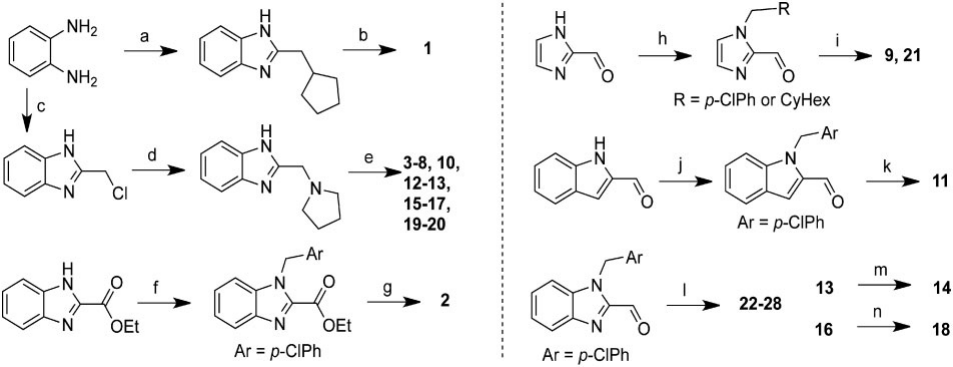
**Synthesis of clemalogues. Summary of synthetic routes used to prepare clemalogues 1–28.** (**a**) cyclopentylacetic acid, PPA, μw, 80°C, 20%; (**b**) ClCH_2_(4-ClPh), K_2_CO_3_, DMF, 60°C, 67%; (**c**) ethyl 4-Cl-3-oxobutanoate, SnCl_2_, EtOH, 80°C; (**d**) pyrrolidine, EtOH, 95°C; 83% for two steps (**e**) Br-CH_2_R, NaH, THF, 0°C to rt, 15–60% or Br-CH_2_R, NaH, TBAI, THF, 0°C to rt, 13–54% or propyl iodide, NaH, THF, 0°C to rt, 47%;(**f**) p-Cl-benzylchloride, NaH, DMF, rt, 54%; (**g**) (i) LiOH, MeOH/water, rt, (ii) pyrrolidine, HATU, DIEA, DMF, rt, 43% over 2 steps; (**h**) p-Cl-benzylchloride, K_2_CO_3_, CH_3_CN, 45°C, 85% or cyclohexylmethyl bromide, K_2_CO_3_, TBAI, CH3CN, 55°C, 30%; (**i**) pyrrolidine, Na(OAc)_3_BH, CH_2_Cl_2_; 30–46%; (**j**) ClCH_2_Ar, K_2_CO_3_, CH_3_CN, 45°C, 44%; (**k**) pyrrolidine, Na(OAc)_3_BH, CH_2_Cl_2_, rt, 65%; (**l**) HNR_2_, Na(OAc)_3_BH, CH_2_Cl_2_, rt, 47–85%; (**m**) H_2_, Pd/C, MeOH, rt, 55%; (**n**) 4 M HCl in dioxane, rt.

To identify a key pharmacophore responsible for suppressing seizure activity in Dravet syndrome zebrafish, we began with structural optimization of the hit compound clemizole hydrochloride, a first-generation antihistamine shown to inhibit seizures in this model (Baraban *et al.*, 2013). We systematically modified both the benzyl and pyrrolidine side chains in clemizole, as well as the benzimidazole core (Fig. 1). Twenty-eight analogues were prepared, in 1–3 steps, using one of the six synthetic routes shown (Fig. 2 and Supplementary Table S1).

Using the established phenotypic-based screening platform (Baraban *et al.*, 2013), and the previously determined concentration range of the parent compound clemizole (Griffin *et al*., 2017), we tested the 28 clemizole analogues for their ability to reduce mean swim velocity of 5 days post-fertilization *scn1lab* mutants at a concentration of 100 µM or 250 µM (six fish per drug treatment). As the initial analogue screening identified non-basic compounds **1** and **2** as toxic, we focused further efforts on analogues that retained the pyrrolidine side chain, or that bore a similar heterocyclic ring with a basic amine. A total of seven such analogues were prepared, among which analogues **22**, **23** and **27** bearing six-membered heterocyclic rings as well as acyclic dimethylamino analogue **24** were found to be toxic. In total, five compounds (17.9%) were identified as toxic at 100 µM, which increased to 12 compounds (42.9%) at 250 µM. Subsequent modification of the 4-chlorophenyl ring, or replacement with cycloalkyl ring surrogates resulted in better tolerance in the zebrafish assay, affording several active analogues and few with any notable toxicity, as detailed below.

**Figure 3:**
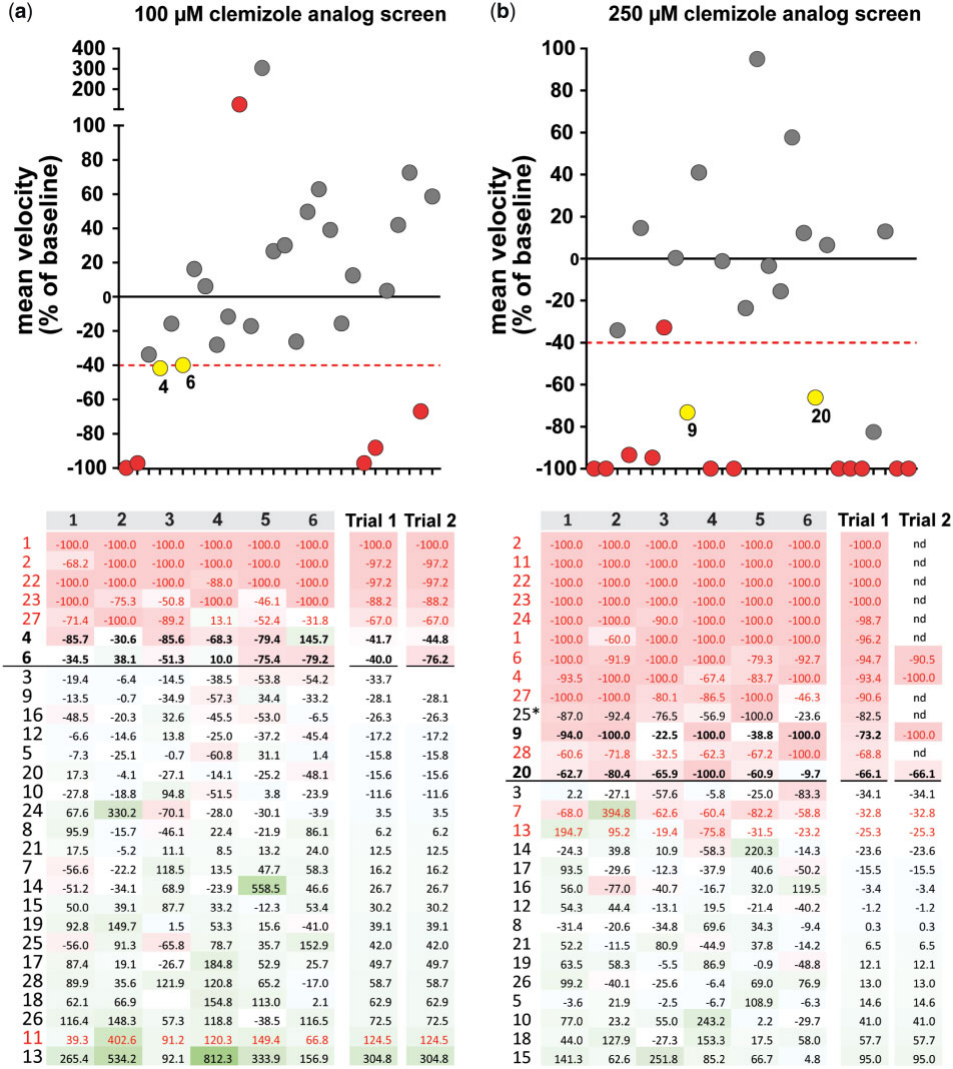
**Phenotypic screening of clemizole analogues.** Twenty-eight clemizole analogues were screened for efficacy in suppressing the high-velocity seizure-like swim behaviour observed in *scn1lab* mutant zebrafish. Plots show the change in mean swim velocity of 5 dpf larvae screened at (**a**) 100 µM, or (**b**) 250 µM. Threshold for inhibition of seizure activity (positive hits—yellow data points) was determined as a reduction in mean swim velocity of ≥40% (red dashed line). The red data points represent compounds that were classified as toxic after 90-min exposure. The heat map shows the % change in velocity for the six individual larva from the first trial (1–6). Mean velocity change from six individual fish is shown for trial 1 and 2. Clemizole analogue **25** (*) failed to go into solution at 250 µM so it was not considered for further testing.

**Figure 4:**
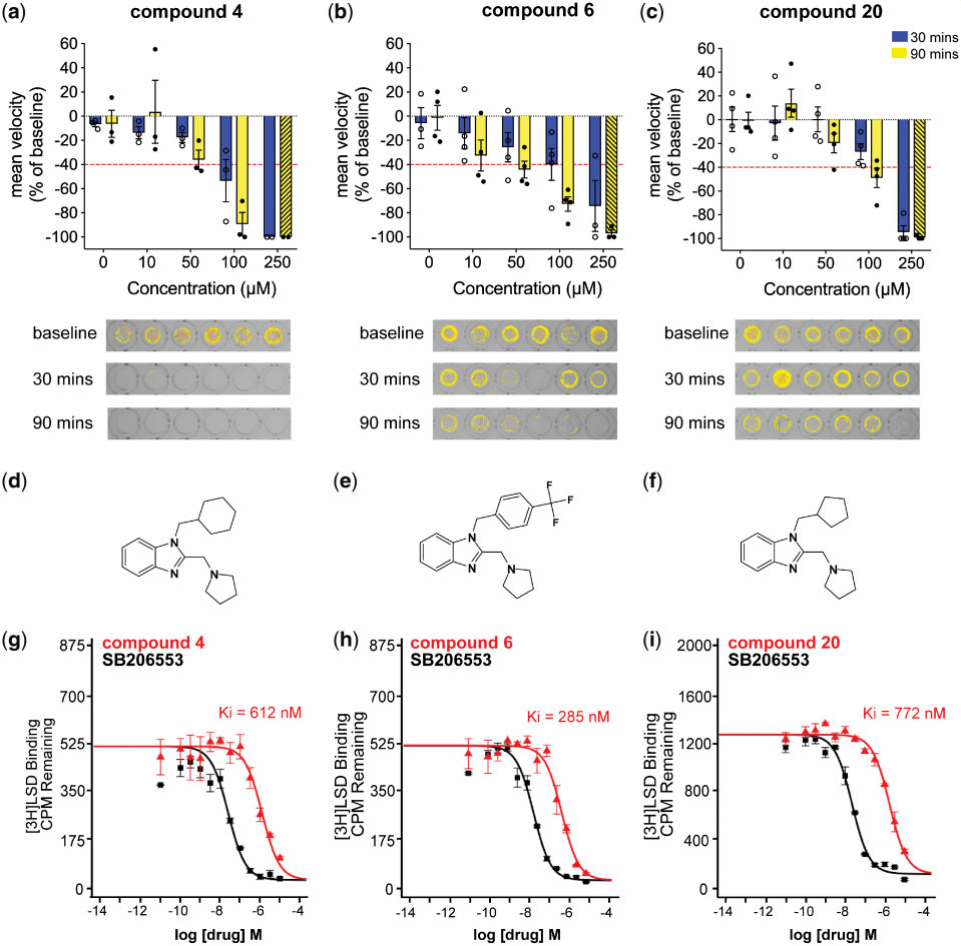
**Evaluation of clemizole analogues that reduce seizure-like swim behaviour in *scn1lab* mutant zebrafish. Clemizole analogues identified as positive from the *in vivo* screen were freshly synthesized and retested for efficacy in suppressing the seizure-like swimming behaviour of 5 dpf *scn1Lab* mutant zebrafish.** Graphs show the change in mean velocity over four concentrations of (**a**) compound **4**, (**b**) compound **6** and (**c**) compound **20.** Each bar represents the mean change in velocity ± SEM from three independent experiments (six individual larva per experiment). Toxicity is indicated by dashed bars. The threshold for a decrease in velocity is ≥40% (red line). Locomotion of larvae was recorded for 10 min after an exposure of 30 min (blue bars) and 90 min (yellow bars). A representative raw 10 min tracking plot is shown for a single experiment of six individual *scn1Lab* zebrafish. The chemical structure for each clemizole analogue is shown (**d–f**). *In vitro* radioligand binding analyses of (**g**) compound **4**, (**h**) compound **6** and (**i**) compound **20** revealed specificity for 5-HT_2B_R over 5-HT_2_R subtypes. SB206553 was used as a positive control for 5-HT_2B_R binding (black). The binding affinity for the other clemizole analogues is given in Supplementary Table S2.

The clemizole analogues **4**, **6**, **9** and **20** were effective in suppressing the *scn1lab* mutant seizure-like behaviour at 100 µM or 250 µM, respectively (Fig. 3). To confirm the anti-seizure effect on convulsive behaviours of *scn1lab* mutants, clemizole analogues **4**, **6**, **9** and **20** were synthesized independently (by Oxygen Healthcare Research Pvt. Ltd.) using the same methodology as described. Newly synthesized compounds were retested at 10, 50, 100 and 250 µM to confirm a concentration-dependent response. Clemizole analogues **4**, **6** and **20** reduced the high-velocity seizure-like swim behaviour observed in the *scn1lab* mutant zebrafish larvae, confirming the initial screening results with analogues synthesized at UCSF (Fig. 4). The resynthesized compound **9** was toxic at 250 µM. This confirmed the result from the second testing of the original compound and suggests the decrease in swim behaviour observed during trial 1 may be a false positive result. Overall, this preliminary structure–activity relationship study revealed the importance of the pyrrolidine side chain in mitigating toxicity and suggests the side chain at N1 of the benzimidazole (or imidazole) core as a promising avenue for further lead optimization of this chemotype.

### Clemizole analogues with anti-seizure activity selectively bind 5-HT_2B_R

From our library of clemizole analogues, we identified three compounds which suppress the convulsive high-velocity swim behaviour observed in the *scn1lab* Dravet syndrome zebrafish model. The parent compound clemizole hydrochloride has previously been reported to have agonist activity for 5-HT_2A_R and 5-HT_2B_R (Griffin *et al.*, 2017). Additionally, 5-HT_2_R modulating compounds exert antiepileptic activity in both preclinical (Sourbron *et al.*, 2016; Griffin *et al.*, 2017) and clinical studies (Griffin *et al.*, 2017; Schoonjans *et al.*, 2017; Kauppila *et al.*, 2018). Therefore, we determined 5-HT_2_R binding affinities for 21 of the clemizole analogues using the radioligand binding assays performed blinded by the NIMH Psychoactive Drug Screening Program (Besnard *et al.*, 2012). Our three hit compounds, **4**, **6** and **20**, had significant preference for 5-HT_2B_R with Ki values of 612 nM, 285 nM and 772 nM, respectively (Fig. 4; Supplementary Table S2). Additionally, these clemizole analogues showed no significant binding to 5-HT_2A_R or 5-HT_2C_R (Ki >10 000 nM). Retrospectively, we observed compounds **5**, **14** and **23** also show selectivity for 5-HT_2B_R with Ki values of 219 nM, 606 nM and 515 nM. In our initial library screen compounds **5** and **14** had no significant effect on swim behaviour and compound **23** was identified as toxic. Additional testing of independently synthesized compound confirmed compounds **5** and **14** have no significant effect on the swim behaviour of the *scn1lab* zebrafish within the constraints of our screening assay (i.e. duration of exposure and compound concentration) (Supplementary Fig. S1). Four of the 21 clemizole analogues (compound **10**, **15**, **17** and **21**) showed no significant binding to any human 5-HT_2_R.

### 5-HT_2B_R agonists suppress seizure activity in the *scn1lab* zebrafish model

**Figure 5:**
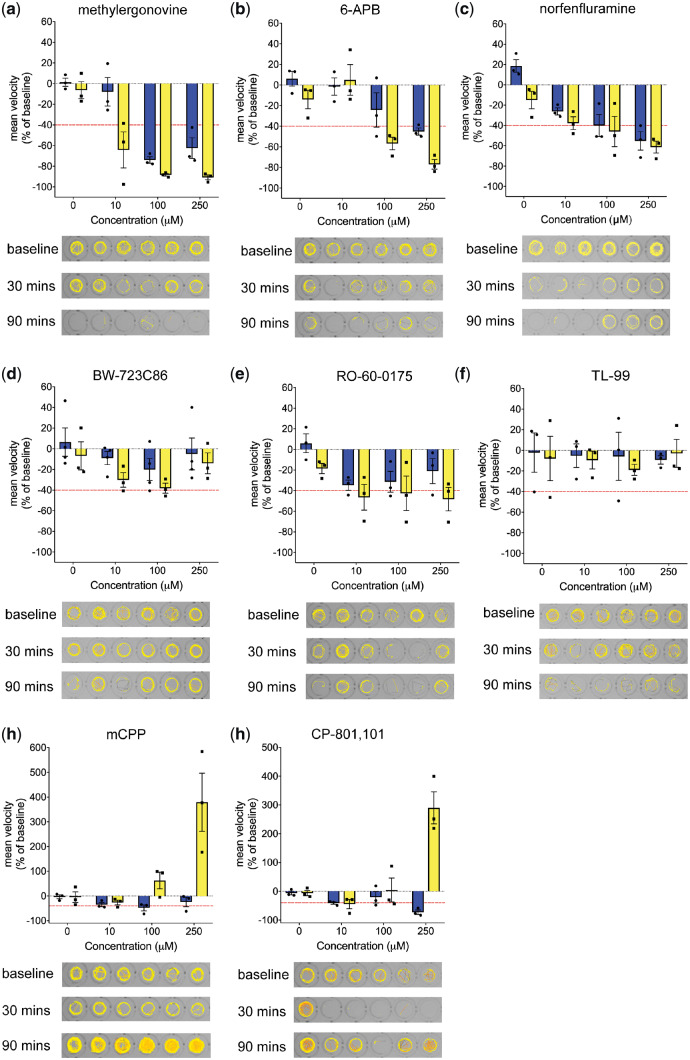
**Dose response evaluation of 5HT2BR agonists in scn1lab mutant zebrafish. 5HT2BR agonists were tested for efficacy in reducing the high-speed seizure-like behaviour in 5 dpf *scn1lab* mutant zebrafish.** Graphs show the change in mean velocity over three concentrations of (**a**) methylergonovine, (**b**) 6-APB, (**c**) norfenfluamine, (**d**) BW-723C86, (**e**) RO-60-0175, (**f**) TL-99, (**g**) m-CPP and (**h**) CP-809, 101. Larvae locomotion was recorded for 10 min after an exposure of 30 min (blue bars) and 90 min (yellow bars). Each bar represents the mean change in velocity ± SEM from three independent experiments (six individual larva per experiment). The threshold for a decrease in velocity is ≥40% (red line). Representative tracking plots of a 10 min recording are shown for six individual 5 dpf *scn1lab* zebrafish at baseline, and following 30 min and 90 min exposure of 100 µM of each compound.

Structure–activity relationship analysis using clemizole analogues suggests 5-HT_2B_R may contribute to the observed anti-seizure activity in *scn1lab* zebrafish. Next, we tested a series of commercially available compounds known to bind 5-HT_2B_R (Roth *et al.*, 2000) for their ability to reduce the seizure-like swim behaviour of the *scn1lab* zebrafish larvae (Supplementary Table S3). Three 5-HT_2B_R agonists, methylergonovine, 6-APB and norfenfluramine suppressed convulsive swim behaviours in a concentration-dependent manner (Fig. 5). Additionally, *scn1lab* mutant larvae treated with 5-HT_2B_R agonist BW-723C86, showed a decrease in seizure-like swim behaviour, but also failed to reach our significance threshold to warrant further testing. This confirms previous observations for the 5-HT_2B_R agonist BW-723C86 (Sourbron *et al.*, 2016). Similarly, the 5-HT_2B_R/5-HT_2C_R agonist Ro60-0175 consistently decreased mean velocity and was borderline effective after 90 min of drug exposure. Treatment with CP-809, 101 gave a biphasic response, as did m-CPP, the active metabolite of trazodone, confirming behavioural responses observed in other model systems (Yonezawa *et al.*, 2008). TL-99, which had the lowest affinity for 5-HT_2_Rs, failed to elicit any behavioural effect in *scn1lab* mutant larvae in our assay (Fig. 5f).

Dopamine receptor agonists with reported 5-HT_2_R were also tested for their ability to reduce seizure-like swim behaviour (Supplementary Fig. S2). Cabergoline, a dopamine agonist with recognized high affinity for activating 5-HT_2B_R (Ki = 1.2 nM) significantly reduced convulsive swim behaviour at 250 µM; however, due to the lack of a concentration-response it did not undergo further testing. Bromocriptine significantly reduced seizure-like swim behaviour at 10 µM; however, toxicity was observed at higher concentrations. Piribedil, a dopamine 2 receptor agonist (Ki = 1.3 nM), also showed toxicity at 100 and 250 µM and the non-selective dopamine agonist, apomorphine, significantly increased mean swim velocity of *scn1lab* mutant larvae, an effect which is also seen in wild-type zebrafish larvae (Ek *et al.*, 2016).

**Figure 6:**
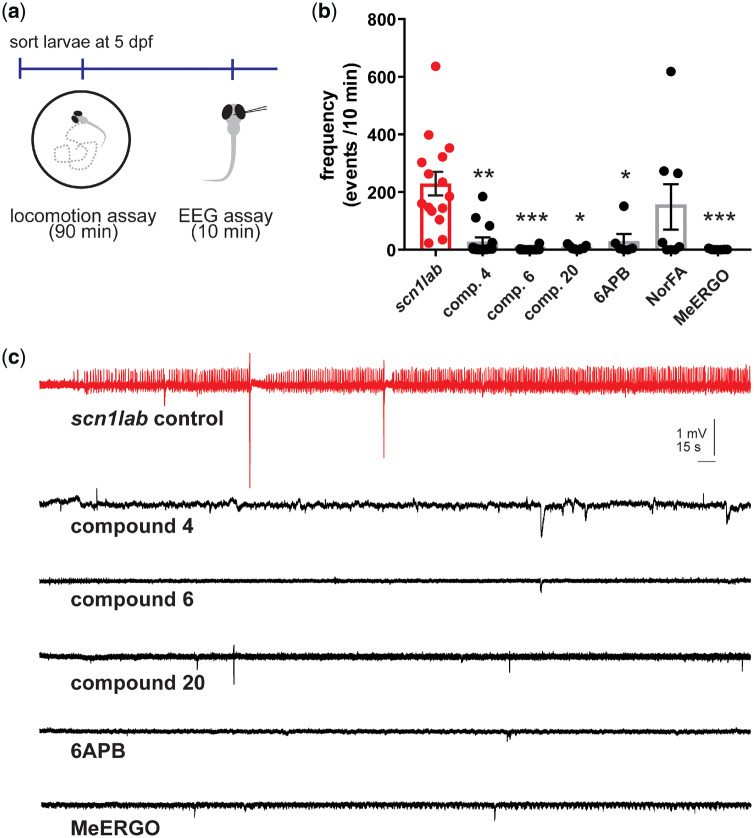
**Electrophysiological assay to identify drugs that rescue the *scn1lab* mutant epilepsy phenotype.** (**a**) Electrophysiology recording were obtained with an electrode placed in the forebrain of 5 dpf agar-immobilized *scn1lab* larvae that had previously showed suppressed seizure-like behaviour in the locomotion assay. (**b**) Bar graphs show the frequency of epileptiform events in a 10 min recording epoch for *scn1lab* larvae exposed to clemizole analogues **4** (*n* = 15), **6** (*n* = 12), **20** (*n* = 9), 6-APB (*n* = 6), norfenfluramine (NorFA) (*n* = 8), methylergonovine (MeERGO) (*n* = 7) or *scn1lab* mutants (*n* = 15). The graph represents mean ± SEM and individual data points are shown. Kruskal–Wallis one-way analysis of variance was used to test for significance. *P <* *0.05; **P <* *0.01; ***P <* *0.001. (**c**) Representative field electrode recording epochs (10 min) are shown for clemizole analogues **4**, **6**, **20**, methylergonovine (MeERGO) and 6-APB. These compounds showed significant changes in the frequency of events compared to untreated *scn1lab* mutant zebrafish (red).

Monitoring electrographic brain activity to confirm seizure suppression is an essential assay to eliminate false positives from behavioural testing (Griffin *et al.*, 2018). By placing a micro-electrode into a visually identified brain region of an agar-immobilized zebrafish larval, stable LFP recordings can be monitored for several hours (Baraban, 2013). At 5 days post-fertilization, LFP recordings of *scn1Lab* zebrafish larvae show an average of 250 abnormal electrographic seizure events during a 10 min recording epoch. LFP recordings of *scn1lab* mutants confirmed significant suppression of electrographic seizure activity after exposure to clemizole analogues **4**, **6** and **20** at 100 µM. Representative LFP recording epochs with only the occasional abnormal electrographic event are shown in Fig. 6. Similarly, 250 µM methylergonovine (*n* = 7; *P *<* *0.001) or 250 µM 6-APB (*n* = 6; *P* = 0.0238) significantly suppressed the frequency of electrographic seizure events in a manner similar to the 5-HT_2B_R selective clemizole analogues **4**, **6** and **20**. Radioligand binding data for methylergonovine, 6-APB and the positively identified clemizole analogues **4**, **6** and **20**, suggest that all five compounds share a binding affinity for 5-HT_2B_R (Table 1).

**Table 1:** Receptor specificity and binding affinity (Ki) for compounds effective in suppressing spontaneous seizure activity in *scn1lab* mutant zebrafish

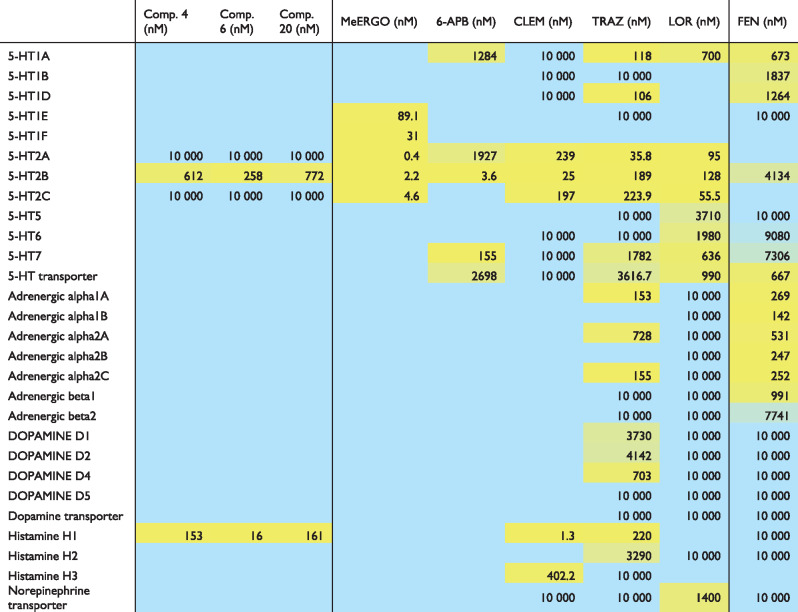

MeERGO, methylergonovine; CLEM, clemizole; TRAZ, trazodone; LOR, lorcaserin; FEN, fenfluramine. Compounds in yellow represent significant binding to the receptor target.

## Discussion

The 5-HT modulating compounds, clemizole, lorcaserin, trazodone and fenfluramine are currently in clinical development for Dravet syndrome. Focusing on clemizole as a promising screening hit, we performed structural–activity–relationship studies to identify targets required for antiepileptic activity. By generating novel clemalogues, we determined that compounds capable of suppressing spontaneous seizures *in vivo* had a binding preference to 5-HT_2B_R. Additionally, we present pharmacological data which suggest that activation of 5-HT_2B_R may be the common antiepileptic mechanism of action shared by the 5-HT modulating compounds showing efficacy in clinical trials and validated preclinical models of Dravet syndrome.

Our approach used an iterative medicinal chemistry process involving the analysis of the chemical structure of clemizole, generating novel analogues of this compound, and using target-engagement assessment through a series of *in vivo* and *in vitro* assays. As a low-cost and efficient alternative to typical experimental animals, this zebrafish-based strategy can be considered a novel disruptive technology in the field of antiepileptic drug discovery. While we are not suggesting that zebrafish replace mammalian models, these findings can be taken as validation of our zebrafish-based approach, as lorcaserin (Belviq; EPX-200), trazodone (EPX-300) and fenfluramine (ZX-008) are already showing clinical anti-seizure efficacy in Dravet syndrome patients (Ceulemans *et al.*, 2012; Ceulemans *et al.*, 2016; Griffin *et al.*, 2017; Schoonjans *et al.*, 2017; Kauppila *et al.*, 2018; Tolete *et al.*, 2018).

Our laboratory has completed blinded phenotype-based screening of over 3500 compounds in a Dravet syndrome zebrafish model (Griffin *et al.*, 2018). This screening platform has spanned multiple drug classes targeting several suggested therapeutic mechanisms. Importantly, a common feature of the small number of compounds identified as capable of suppressing spontaneous seizure activity in this assay (<0.2% of all compounds screened; i.e. clemizole, lorcaserin and trazodone), appears to be a binding affinity for 5HT_2_R subtypes. Here, we identified three novel clemizole analogues and two additional commercially available compounds that mimic the suppression of seizure activity seen previously. These findings support a working hypothesis that targeting a combination of, or a single, 5HT_2_R is therapeutic for Dravet syndrome. Similar to how most of the present antiepileptic drugs have been discovered and studied, these findings are limited to the pharmacological tools available and will, ultimately, require molecular and/or functional strategies to precisely confirm a mechanism of action. Nonetheless, our conclusion is consistent with pharmacological and knockout mouse studies implicating 5-HT_2_ receptors in anti-seizure and anti- Sudden Unexplained Death in Epilepsy actions (Tupal and Faingold, 2006).

The structural similarity for 5-HT_2_ receptors makes developing therapeutic compounds with high receptor subtype specificity and affinity challenging. In comparison to the parent compound clemizole, our clemizole analogues gained 5-HT_2_ receptor specificity but decreased receptor affinity. Therefore, these analogues provide a useful tool for understanding the mechanism of the anti-seizure action of clemizole. In addition to receptor subtype similarity, 5-HT_2_R agonists may have functional selectivity, whereby, a ligand can preferentially activate one receptor-linked intracellular signalling pathway (i.e., Gq-linked calcium flux or β-arrestin recruitment). While the signalling bias of each clemizole analogue remains to be determined, methylergonovine is known to have a strong preference for activating the β-arrestin pathway (Wacker *et al.*, 2013). As methylergonovine is capable of reducing seizures in the *scn1lab* larvae, functional signalling may be an additional consideration when determining the mechanism of antiepileptic action shared between these serotonin modulating compounds.

Within the field of drug development, 5-HT_2B_R agonist activity has sometimes been perceived as negative due to the putative involvement of this receptor subtype in heart valve pathogenesis (Roth, 2007; Papoian *et al.*, 2017). Indeed, activation of 5-HT_2B_R can induce a mitogenic effect on valvular endothelial cells and has resulted in the discontinued development of some compounds (Roth, 2007). Perhaps one of the most recognizable compounds to be withdrawn under Food and Drug Administration recommendation is the 5-HT reuptake blocker fenfluramine (Rothman *et al.*, 2000), a compound currently in clinical trial as an add-on therapy for Dravet syndrome patients with, as yet, no reported negative cardiovascular events (Ceulemans *et al.*, 2012; Ceulemans *et al.*, 2016). Once approved as an anorectic, it was later removed from the market after heart valve defects were noted in ∼25% of patients (Abenhaim *et al.*, 1996; Khan *et al.*, 1998). Norfenfluramine, the active metabolite of fenfluramine is a potent activator of 5-HT_2B_R and may contribute to these observed heart valve defects (Fitzgerald *et al.*, 2000). In the *scn1lab* zebrafish Dravet syndrome model, fenfluramine is effective in suppressing spontaneous seizures (Dinday and Baraban, 2015; Sourbron *et al.*, 2016), additionally, norfenfluramine was able to restore the swimming behaviour or *scn1lab* mutant larvae to wild-type levels (Stage I) and reduce spontaneous seizure events in some, but not all larvae (5 out of 8 larvae; Fig. 6) suggesting that this active metabolite may contribute to the anti-seizure activity of fenfluramine.

Our previous working hypothesis proposed that clemizole, lorcaserin, trazodone and fenfluramine could increase gamma-aminobutyric acid (GABA)-mediated synaptic inhibition through direct activation of (i) 5-HT_2A_Rs, which are expressed throughout the central nervous system; or (ii) 5-HT_2C_Rs which are expressed on a subpopulation of inhibitory interneurons (Liu *et al.*, 2007). However, newly synthesized clemalogues with selective affinity for 5-HT_2A_ or 5-HT_2C_ receptor subtypes were not consistently observed to inhibit spontaneous seizures in our zebrafish model whereas those with 5-HT_2B_R binding properties were effective. The histamine receptor (H1) is also a common site of action for clemizole (a first-generation antihistamine) and many of the compounds described in this manuscript. This receptor was excluded as the mechanism of action site because antihistamines are often contraindicated as anti-seizure medications in paediatric epilepsies (Miyata *et al.*, 2011). Additionally, prior screening of more than 40 different H1-receptor binding compounds failed to identify any other antihistamines with anti-seizure activity (Griffin *et al.*, 2017).

Zebrafish have a single 5-HT_2B_R orthologue, which has 62.0% protein identity compared to the human protein (Griffin *et al.*, 2017). While *htr2b* is expressed in the zebrafish brain (Theodoridi *et al.*, 2017), the precise cell type in the larval central nervous system where this receptor is expressed has not been established. In the mammalian brain, prominent central nervous system expression of 5-HT_2B_R has been reported on astrocytes (Sanden *et al.*, 2000; Zhang *et al.*, 2010), and receptor activation can result in elevated calcium signalling. 5-HT_2_R mediated enhancement of astrocyte signalling could (i) release glutamate onto GABAergic interneurons causing a potentiation of inhibitory interneuron signalling (Kang *et al.*, 1998), or (ii) provide positive feedback autoregulation to somatostatin-expressing GABAergic interneurons innervating the dendrites of excitatory pyramidal neurons (Matos *et al.*, 2018). Additionally, neuronal 5HT_2B_R expression has been reported in Purkinje cells (Choi and Maroteaux, 1996) and neurons in the dorsal raphe nuclei (Diaz *et al.*, 2012). Optogenetic stimulation of these raphe neurons has been shown to suppress both hippocampal and cortical neuronal activity, as well as Sudden Unexplained Death in Epilepsy in mice (Wang *et al.*, 2015; Lottem *et al.*, 2016; Zhang *et al.*, 2018). There is also evidence to suggest that 5-HT_2B_ receptors directly modulate serotonin levels independently of the serotonin transporter (Hertz *et al.*, 2015). Finally, published evidence for 5-HT_2B_R expression on microglia (Krabbe *et al.*, 2012; Kolodziejczak *et al.*, 2015) could suggest a modulatory role as part of the tripartite synapse (Ji *et al.*, 2013). Each of these possibilities are presented as potential mechanisms of action for discussion purposes only and merit further investigation using the pharmacological tools developed here.

In conclusion, our results imply a 5-HT_2B_R mechanism of action for several serotonergic compounds currently in clinical development for Dravet syndrome and suggest that a zebrafish-based strategy may represent a promising avenue to the discovery and development of drugs for treating catastrophic epilepsies of childhood.

## Supplementary Material

fcz008_Supplementary_Materials

## Abbreviations

AEDs: antiepileptic drugs
dpf: days post-fertilization
FDA: Food and Drug Administration
SAR: structure–activity relationship
SUDEP: Sudden Unexplained Death in Epilepsy
5-HT: serotonin
5-HTR: serotonin receptor
