# Supplementary material for: Zebrafish studies identify serotonin receptors mediating antiepileptic activity in Dravet syndrome
Source: Brain Commun. 2019 Aug 1;1(1):fcz008. doi: 10.1093/braincomms/fcz008 (PMC6798786; doi:10.1093/braincomms/fcz008)
Supplement: fcz008_Supplementary_Materials [file fcz008_supplementary_materials.zip › fcz008_Supplementary_Methods.pdf]

Supplementary Methods

Clemizole analog synthesis

1-[(4-Chlorophenyl)methyl]-2-(cyclopentylmethyl)-1H-benzimidazole (1)

Step 1: A mixture of *o*-phenylenediamine (0.25 g, 2.3 mmol), cyclopentylacetic acid (0.29 ml, 2.3 mmol) and polyphosphoric acid (1.0 ml) were heated in a microwave reactor at 80 ^{o}C for 30 minutes. The reaction mixture was diluted with ethyl acetate, washed with aqueous saturated sodium bicarbonate solution, water and brine. The organic layer was dried over magnesium sulfate, concentrated and purified by flash column chromatography (50% ethyl acetate/hexanes) to obtain 2-(cyclopentylmethyl)- 1H-benzimidazole as a light brown solid (93 mg, 20% yield). ^{1}H NMR (400 MHz, CDCl3) δ 7.57 (dd, J = 6.1, 3.2 Hz, 2H), 7.29 – 7.19 (m, 2H), 2.95 (d, J = 7.5 Hz, 2H), 2.48-2.38 (m, 1H), 1.96 – 1.78 (m, 2H), 1.72 – 1.50 (m, 4H), 1.36 – 1.23 (m, 2H); LC-MS (m/z) for $C_{13}H_{17}N_2^+$ [M+H]$^+$: calculated 201.13, found 200.99.

Step 2: 4-Chlorobenzyl chloride (0.04 g, 0.2 mmol) was added to a mixture of 2-(cyclopentylmethyl)-1H-benzimidazole (0.05 g, 0.2 mmol) and potassium carbonate (0.069 g, 0.5 mmol) in N,N-dimethylformamide (2 mL). After stirring at 60 ^{o}C for 3h, the reaction mixture was diluted with ethyl acetate, washed with water and brine. The organic layer was dried over magnesium sulfate, concentrated and purified by flash column chromatography (25% ethyl acetate/hexanes) to obtain 1-[(4-chlorophenyl)methyl]-2-(cyclopentylmethyl)- 1H-benzimidazole (**1**) as a white solid (55mg, 67% yield). ^{1}H NMR (400 MHz, CDCl3) δ 7.79 (d, J = 7.8 Hz, 1H), 7.33 – 7.15 (m, 5H), 6.98 (d, J = 8.3 Hz, 2H), 5.35 (s, 2H), 2.85 (d, J = 7.5 Hz, 2H), 2.51 – 2.39 (m, 1H), 1.85 (td, J = 11.5, 6.9 Hz, 2H), 1.73 – 1.53 (m, 4H), 1.35 – 1.22 (m, 2H); LC-MS (m/z) $C_{20}H_{22}ClN_2^+$ [M+H]$^+$: calculated 325.14, found 324.98.

1-[(4-Chlorophenyl)methyl]-2-(pyrrolidine-1-carbonyl)-1H-benzimidazole (2)

Step 1: A mixture of 1H-benzimidazole-2-carboxylic acid ethyl ester (0.1 g, 0.5 mmol) and pyrrolidine (0.216 ml, 2.6 mmol) were heated to 130 °C in the microwave reactor for 30 minutes. The reaction mixture was concentrated and azeotrope dried with toluene to obtain about 115 mg of 2-(pyrrolidine-1-carbonyl)-1H-benzimidazole as a brown solid which was used without further purification.

Step 2: 4-Chlorobenzyl chloride (0.03 g, 0.2 mmol) was added to a mixture of sodium hydride, 60% (0.005 g, 0.2 mmol) and 2-(pyrrolidine-1-carbonyl)-1H-benzimidazole (0.04 g, 0.2 mmol,) in N,N-dimethylformamide (1 mL). After stirring at room temperature for 3 h, the reaction mixture was quenched with aqueous saturated ammonium chloride and extracted with ethyl acetate. The organic extracts were washed with water and brine. The organic layer was dried over magnesium sulfate, concentrated and purified by flash column chromatography (0-50% ethyl acetate/hexanes) to obtain 1-[(4-chlorophenyl)methyl]-2-(pyrrolidine-1-carbonyl)-1H-benzimidazole (**2**) as a clear oil (29 mg, 46% yield). ^{1}H NMR (400 MHz, CDCl3) δ 7.89 – 7.82 (m, 1H), 7.37 – 7.24 (m, 7H), 5.71 (s, 2H), 3.90 (br t, J = 6.0 Hz, 2H), 3.67 (br t, J = 6.3 Hz, 2H), 2.01 – 1.90 (m, 4H); LC-MS (m/z) for $C_{19}H_{19}ClN_3O^+$ $[M+H]^+$: calculated 340.12, found 340.21.

1-(Cyclohexylmethyl)-2-[(pyrrolidin-1-yl)methyl]-1H-benzimidazole (4)

Step 1: Tin(II) chloride, dihydrate (0.209 g, 0.9 mmol) was added to a mixture of *o*-phenylenediamine (1.0 g, 9.2 mmol) and ethyl 4-chloro-3-oxobutanoate (1.25 ml, 9.2 mmol) in ethanol (20 mL) and heated to 80 °C for 2 h. The reaction mixture was concentrated to remove

ethanol, washed with hexanes and dried to obtain about 1.7 g of crude 2-(chloromethyl)-1H-benzimidazole as a yellow solid which was used without further purification.

Step 2: A mixture of 2-(chloromethyl)-1H-benzimidazole (1.5 g, 9.0 mmol) and pyrrolidine (14.8 ml, 180.1 mmol) in ethanol (20 mL) was heated to 95 ^{o}C for 2h and room temperature for 72 h. The reaction mixture was concentrated and purified by flash column chromatography (0-10% methanol/dichloromethane with 10% 7 N ammonia in methanol) to obtain 2-[(pyrrolidin-1-yl)methyl]-1H-benzimidazole as a reddish-brown solid (1.5 g, 83% yield). ^{1}H NMR (400 MHz, CDCl3) δ 7.65 – 7.53 (m, 2H), 7.31 – 7.20 (m, 2H), 4.17 (s, 2H), 3.50 (s, 2H), 2.94 – 2.83 (m, 4H), 1.93 (br s, 4H); LC-MS (m/z) for $C_{12}H_{16}N_3^+$ $[M+H]^+$: calculated 202.13, found 202.07.

Step 3: Cyclohexylmethyl bromide (0.038 ml, 0.3 mmol) and tetrabutylammonium iodide (0.009 g, 0.025 mmol) were added to a cooled (0 ^{o}C) mixture of 2-[(pyrrolidin-1-yl)methyl]-1H-benzimidazole (0.05 g, 0.25 mmol) and sodium hydride, 60% (0.007 g, 0.3 mmol) in tetrahydrofuran (1 mL). The reaction mixture was heated to 50 ^{o}C for 2 h and then diluted with ethyl acetate, washed with 10% aqueous ammonium hydroxide solution, water and brine. The organic layer was dried over magnesium sulfate, concentrated and purified by flash column chromatography (5% methanol/dichloromethane) to obtain 1-(cyclohexylmethyl)-2-[(pyrrolidin-1-yl)methyl]-1H-benzimidazole (**4**) as a reddish-brown oil (39 mg, 52% yield). ^{1}H NMR (400 MHz, CDCl3) δ 7.85 – 7.68 (m, 1H), 7.38 – 7.20 (m, 3H), 4.17 (d, J = 7.3 Hz, 2H), 3.95 (s, 2H), 2.66 – 2.54 (m, 4H), 1.96 – 1.63 (m, 10H), 1.30 – 1.03 (m, 5H); LC-MS (m/z) for $C_{19}H_{28}N_3^+$ $[M+H]^+$: calculated 298.22, found 298.06.

2-[(Pyrrolidin-1-yl)methyl]-1-{[4-(trifluoromethyl)phenyl]methyl}-1H-benzimidazole (6)

4-(Trifluoromethyl)benzyl bromide (0.038 ml, 0.2 mmol) was added to a cooled (0 ^{o}C) mixture of 2-[(pyrrolidin-1-yl)methyl]-1H-benzimidazole (0.05 g, 0.2 mmol) and sodium hydride, 60% (0.007 g, 0.3 mmol) in tetrahydrofuran (1 mL). The reaction mixture was stirred at room temperature for 2h and then diluted with ethyl acetate, washed with 10% aqueous ammonium hydroxide solution, water and brine. The organic layer was dried over magnesium sulfate, concentrated and purified by flash column chromatography (5% methanol/dichloromethane) & (50% acetone/dichloromethane) to obtain 2-[(pyrrolidin-1-yl)methyl]-1-{[4-(trifluoromethyl)phenyl]methyl}-1H-benzimidazole (**6**) as a pale yellow oil (25 mg, 28% yield) that solidifies on standing. ^{1}H NMR (400 MHz, CDCl3) δ 7.81 (d, J = 7.3 Hz, 1H), 7.57 (d, J = 8.3 Hz, 2H), 7.34 – 7.18 (m, 5H), 5.67 (s, 2H), 3.90 (s, 2H), 2.61 – 2.49 (m, 4H), 1.77 – 1.61 (m, 4H); LC-MS (m/z) for $C_{20}H_{21}F_{3}N_{3}^{+}$ $[M+H]^{+}$: calculated 360.16, found 360.05.

1-[(4-Chlorophenyl)methyl]-2-[(pyrrolidin-1-yl)methyl]-1H-imidazole (9)

Step 1: 1H-Imidazole-2-carbaldehyde (0.25 g, 2.6 mmol), 4-chlorobenzyl chloride (0.5 g, 3.1 mmol) & potassium carbonate (0.72 g, 5.2 mmol) were stirred in acetonitrile (20 mL) at 45 ^{o}C for 18h. The reaction mixture was diluted with ethyl acetate, washed with water and brine. The organic layer was dried over magnesium sulfate, concentrated and purified by flash column chromatography (50% ethyl acetate/hexanes) to obtain 1-[(4-chlorophenyl)methyl]-1H-imidazole-2-carbaldehyde as a pale green oil (0.49 g, 85% yield). ^{1}H NMR (400 MHz, CDCl3) δ 9.82 (s, 1H), 7.32 – 7.26 (m, 3H), 7.16 – 7.10 (m, 3H), 5.56 (s, 2H); LC-MS (m/z) for $C_{11}H_{10}ClN_{2}O^{+}$ $[M+H]^{+}$: calculated 221.04, found 220.83.

Step 2: Sodium triacetoxyborohydride (0.053 g, 0.2 mmol) was added to a mixture of 1-[(4-chlorophenyl)methyl]-1H-imidazole-2-carbaldehyde (0.05 g, 0.2 mmol) and pyrrolidine (0.019 ml, 0.2 mmol) stirring in dichloromethane (2 mL). After stirring at room temperature for 18 h, the reaction mixture was washed with 10% aqueous ammonium hydroxide solution, water and brine. The organic layer was dried over magnesium sulfate, concentrated and purified by flash column chromatography (0-10% methanol/dichloromethane/10% 7N ammonia in methanol) to obtain 1-[(4-chlorophenyl)methyl]-2-[(pyrrolidin-1-yl)methyl]-1H-imidazole (**9**) as a yellow solid (19 mg, 30% yield). ^{1}H NMR (400 MHz, CDCl3) δ 7.36 – 7.26 (m, 2H), 7.08 (d, J = 8.3 Hz, 2H), 6.99 (d, J = 1.2 Hz, 1H), 6.86 (d, J = 1.2 Hz, 1H), 5.27 (s, 2H), 3.67 (s, 2H), 2.55 – 2.46 (m, 4H), 1.85 – 1.67 (m, 4H); LC-MS (m/z) for $C_{15}H_{19}ClN_3^+$ $[M+H]^+$: calculated 276.12, found 276.01.

1-[(4-Chlorophenyl)methyl]-2-[(pyrrolidin-1-yl)methyl]-1H-indole (11)

Step 1: 4-Chlorobenzyl chloride (0.133 g, 0.8 mmol) was added to a mixture of 1H-indole-2-carbaldehyde (0.1 g, 0.7 mmol) and potassium carbonate (0.19 g, 1.4 mmol) in acetonitrile (5 mL). After stirring at 45 ^{o}C for 18 h, the reaction mixture was diluted with ethyl acetate, washed with water and brine. The organic layer was dried over magnesium sulfate, concentrated and purified by flash column chromatography (25% ethyl acetate/hexanes) to obtain 1-[(4-chlorophenyl)methyl]-1H-indole-2-carbaldehyde as an orange colored solid (81 mg, 43% yield). ^{1}H NMR (400 MHz, CDCl3) δ 9.92 (s, 1H), 7.80 (d, J = 7.8 Hz, 1H), 7.45 – 7.35 (m, 3H), 7.30 – 7.19 (m, 3H), 7.05 (d, J = 8.5 Hz, 2H), 5.82 (s, 2H); LC-MS (m/z) for $C_{16}H_{13}ClNO^+$ $[M+H]^+$: calculated 270.06, found 269.96.

Step 2: Sodium triacetoxyborohydride (0.043 g, 0.2 mmol) was added to a cooled (0 °C) mixture of 1-[(4-chlorophenyl)methyl]-1H-indole-2-carbaldehyde (0.05 g, 0.2 mmol) and pyrrolidine (0.015 ml, 0.2 mmol) in dichloromethane. After stirring at room temperature for 18 h, the reaction mixture was washed with 10% aqueous ammonium hydroxide solution, water and brine. The organic layer was dried over magnesium sulfate, concentrated and purified by flash column chromatography (50% ethyl acetate/hexanes) to obtain 1-[(4-chlorophenyl)methyl]-2-[(pyrrolidin-1-yl)methyl]-1H-indole (**11**) as a pale yellow oil (39 mg, 65% yield). ^{1}H NMR (400 MHz, CDCl3) δ 7.70 – 7.51 (m, 1H), 7.32 – 7.09 (m, 5H), 6.96 (d, J = 8.3 Hz, 2H), 6.46 (s, 1H), 5.53 (s, 2H), 3.68 (s, 2H), 2.58 – 2.41 (m, 4H), 1.80 – 1.66 (m, 4H); LC-MS (m/z) for $C_{20}H_{22}ClN_2^+$ $[M+H]^+$: calculated 325.14, found 325.02.

1-(Cyclopentylmethyl)-2-[(pyrrolidin-1-yl)methyl]-1H-benzimidazole (20)

Bromomethyl cyclopentane (0.092 ml, 0.7 mmol) and tetrabutylammonium iodide (0.009 g, 0.02 mmol) were added to a mixture of 2-[(pyrrolidin-1-yl)methyl]-1H-benzimidazole (0.05 g, 0.2 mmol) and sodium hydride, 60% (0.009 g, 0.4 mmol) in N,N-dimethylformamide (1 mL). After stirring at 55 °C for 18h, the reaction mixture was diluted with ethyl acetate, washed with water and brine. The organic layer was dried over magnesium sulfate, concentrated and purified by flash column chromatography (0-10% methanol/dichloromethane) to obtain 1-(cyclopentylmethyl)-2-[(pyrrolidin-1-yl)methyl]-1H-benzimidazole (**20**) as a reddish brown oil (35 mg, 50% yield). ^{1}H NMR (400 MHz, CDCl3) δ 7.85 – 7.66 (m, 1H), 7.41 – 7.22 (m, 3H), 4.28 (d, J = 7.8 Hz, 2H), 3.96 (s, 2H), 2.64 – 2.44 (m, 5H), 1.83 – 1.54 (m, 10H), 1.42 – 1.24 (m, 2H); LC-MS (m/z) for $C_{18}H_{26}N_3^+$ $[M+H]^+$: calculated 284.21, found 284.01.

({1-[(4-Chlorophenyl)methyl]-1H-1,3-benzodiazol-2-yl}methyl)diethylamine (24)

Sodium triacetoxyborohydride (0.043 g, 0.2 mmol) was added to a mixture of 1-(4-chlorobenzyl)-1H-benzimidazole-2-carbaldehyde (0.05 g, 0.18 mmol) and diethylamine (0.019 mL, 0.18 mmol) in dichloromethane (2 mL). After stirring at room temperature for 18 h, the reaction mixture was washed with water and brine. The organic layer was dried over magnesium sulfate, concentrated and purified by flash column chromatography (0-100% ethyl acetate/hexanes) to obtain ({1-[(4-chlorophenyl)methyl]-1H-1,3-benzodiazol-2-yl}methyl)diethylamine (**24**) as a reddish-brown oil (50 mg, 82% yield). ^{1}H NMR (400 MHz, CDCl3) δ 7.80 (d, J = 7.6 Hz, 1H), 7.33 – 7.17 (m, 5H), 7.02 (d, J = 8.8 Hz, 2H), 5.64 (s, 2H), 3.84 (s, 2H), 2.57 (q, J = 7.1 Hz, 4H), 1.00 (t, J = 7.2 Hz, 6H); LC-MS (m/z) for $C_{19}H_{23}ClN_3^+$ $[M+H]^+$: calculated 328.15, found 327.98.

***tert*-Butyl 4-({1-[(4-chlorophenyl)methyl]-1H-benzimidazol-2-yl}methyl)piperazine-1-carboxylate (25)**

Sodium triacetoxyborohydride (0.043 g, 0.2 mmol) was added to a mixture of 1-(4-chlorobenzyl)-1H-benzimidazole-2-carbaldehyde (0.05 g, 0.18 mmol) and *tert*-butyl 1-piperazinecarboxylate (0.034 g, 0.18 mmol) in dichloromethane (2 mL). After stirring at room temperature for 18 h, the reaction mixture was washed with water and brine. The organic layer was dried over magnesium sulfate, concentrated and purified by flash column chromatography (0-100% ethyl acetate/hexanes) to obtain *tert*-butyl 4-({1-[(4-chlorophenyl)methyl]-1H-benzimidazol-2-yl}methyl)piperazine-1-carboxylate (**25**) as a foamy pale yellow oil (59 mg, 72% yield). ^{1}H NMR (400 MHz, CDCl3) δ 7.79 (d, J = 7.4 Hz, 1H), 7.34 – 7.19 (m, 6H), 7.03 (d, J = 8.3 Hz, 2H), 5.55

(s, 2H), 3.77 (s, 2H), 3.33 (br s, 4H), 2.45 (br s, 4H), 1.46 (s, 9H); LC-MS (m/z) for $C_{24}H_{30}ClN_4O_2^+$ $[M+H]^+$: calculated 441.20, found 441.08.
