# Supplementary material for: Zebrafish studies identify serotonin receptors mediating antiepileptic activity in Dravet syndrome
Source: Brain Commun. 2019 Aug 1;1(1):fcz008. doi: 10.1093/braincomms/fcz008 (PMC6798786; doi:10.1093/braincomms/fcz008)
Supplement: fcz008_Supplementary_Materials [file fcz008_supplementary_materials.zip › fcz008_Supplementary_Material_Figures_Tables.pdf]

Supplementary Figures

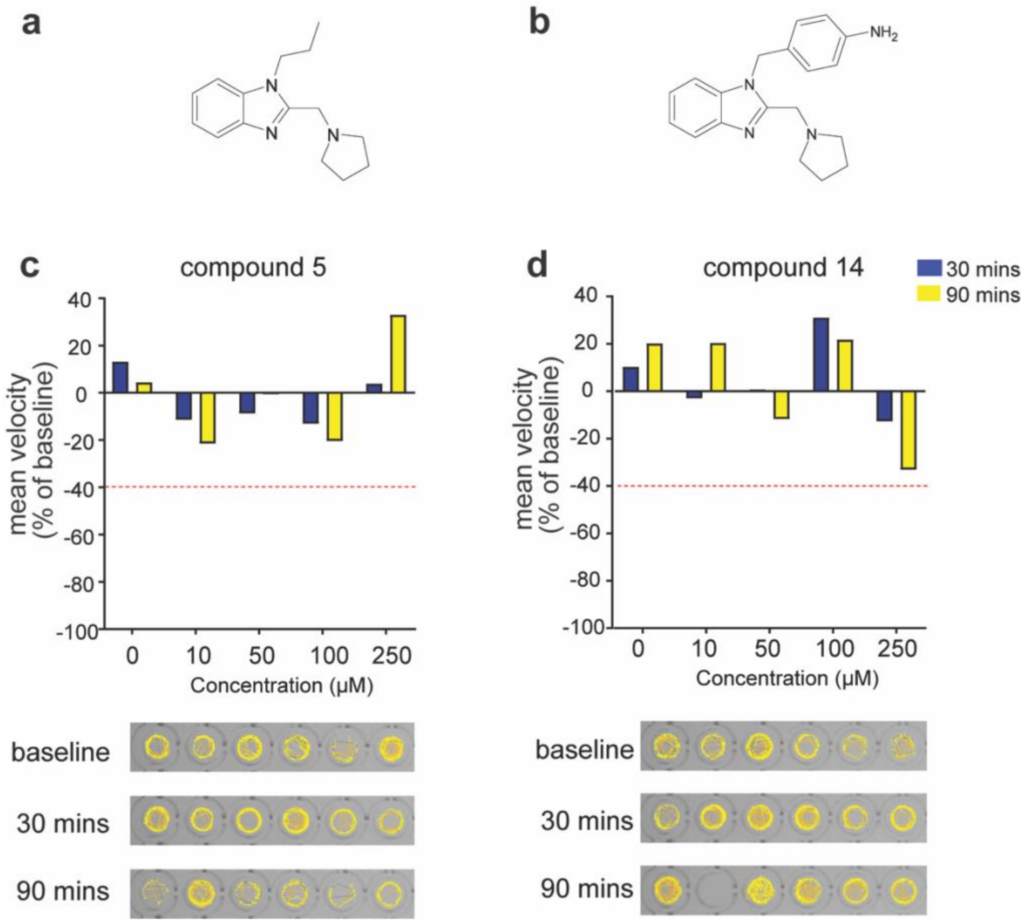

Figure S1: Behavioral screening of resynthesized clemizole analogs 5 and 14. Clemizole analogs (**a**) **5** and (**b**) **14** were identified as having specific binding for $5HT_{2B}R$. Subsequently, they were independently synthesized and tested. Behavioral testing confirmed the previous screening results and showed no significant effect on the high-speed seizure-like behavior in 5 dpf *scn1lab* mutant zebrafish. Graphs show the change in mean velocity of six fish treated with each clemalog (**c,d**). The threshold for a decrease in velocity is ≥ 40% (red line). Locomotion of larvae was recorded for 10 min after an exposure of 30 min (blue bars) and 90 min (yellow bars). The raw 10 min tracking plot is shown for the baseline, 30 min and 90 min exposure of 100 µM.

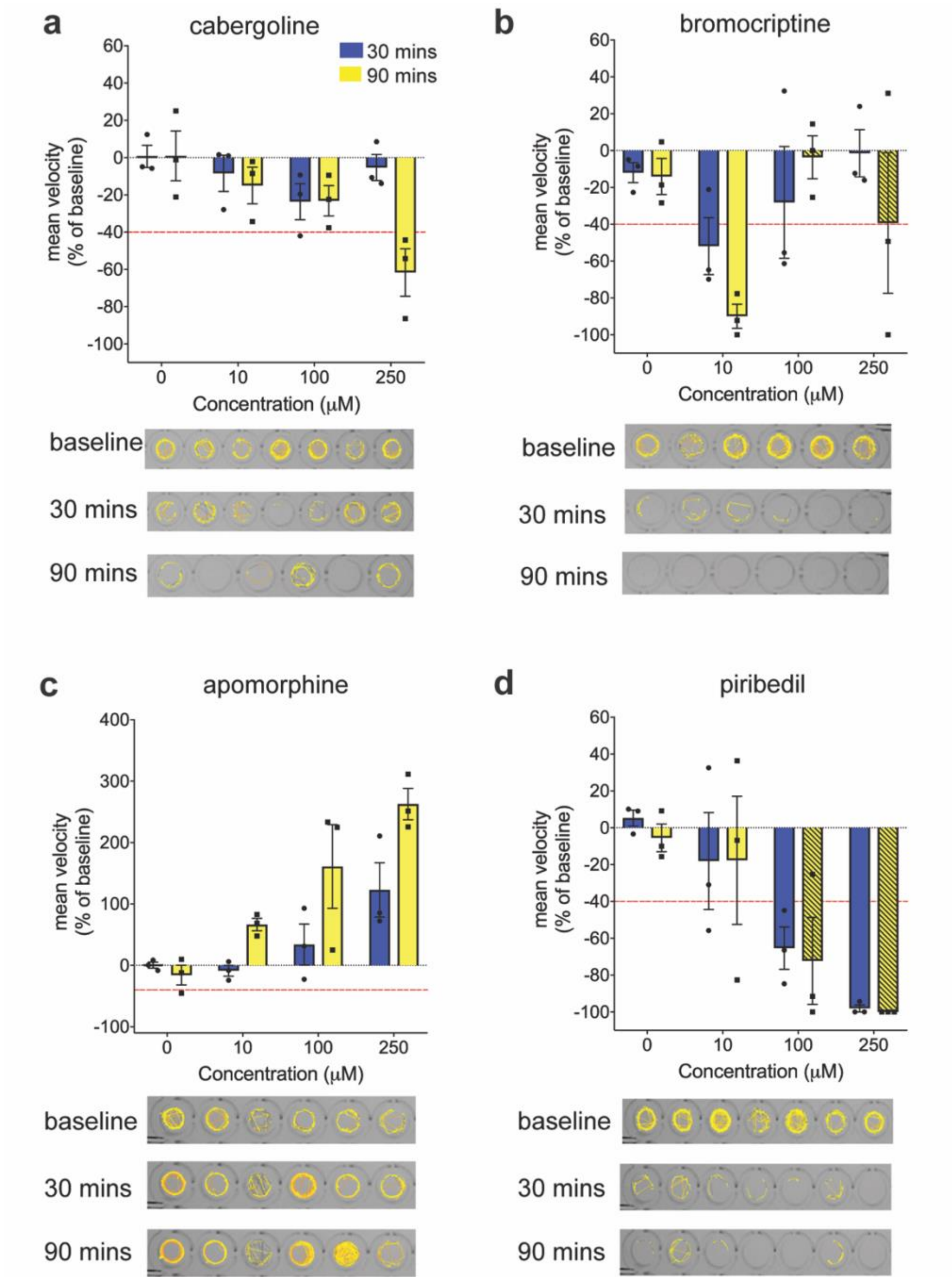

Figure S2: Dose response evaluation of $5HT_{2B}R$ agonists in *scn1lab* mutant zebrafish. $5HT_{2B}R$ agonists were tested for efficacy in reducing the high-speed seizure-like behavior in 5 dpf *scn1lab* mutant zebrafish. Graphs show the change in mean velocity over three concentrations of (**a**) cabergoline, (**b**) bromocriptine, (**c**) apomorphine, and (**d**) piribedil. Larvae locomotion was recorded for 10 min after an exposure of 30 min (blue bars) and 90 min (yellow bars). Each bar represents the mean change in velocity ± SEM from three independent experiments (six individual

larva per experiment). The threshold for a decrease in velocity is ≥ 40% (red line). Toxicity is indicated by dashed bars. Representative tracking plots of a 10 min recording are shown for six individual 5 dpf *scn1lab* zebrafish at baseline and following 30 min and 90 min exposure of 100 µM of each compound.

Supplementary Tables

Table S1: Clemizole analogs library

Compound	Structure	MW
**1**	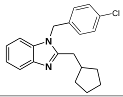	324.85
**2**	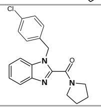	339.82
**3**	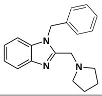	337.42
**4**	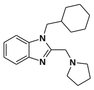	297.44
**5**	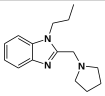	289.37
**6**	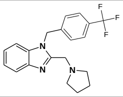	359.39
**7**	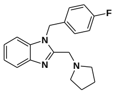	309.38
**8**	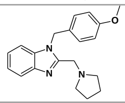	367.44
**9**	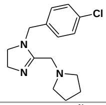	275.78
**10**	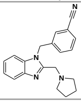	316.40
**11**	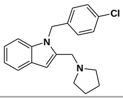	324.85
**12**	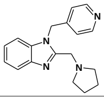	384.43
**13**	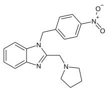	336.39
**14**	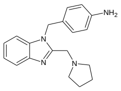	306.40

Compound	Structure	MW
**15**	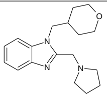	299.41
**16**	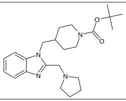	398.54
**17**	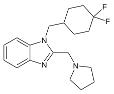	333.42
**18**	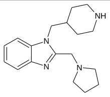	371.35
**19**	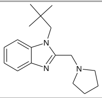	317.43
**20**	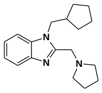	283.41
**21**	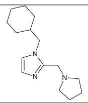	339.43
**22**	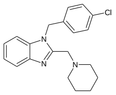	339.86
**23**	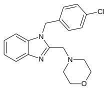	341.83
**24**	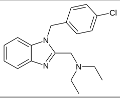	327.85
**25**	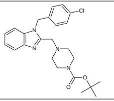	440.97
**26**	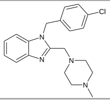	354.88
**27**	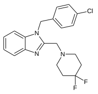	375.84
**28**	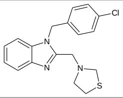	343.87

Table S2: Clemizole analogs binding affinity (Ki) to human 5-HT_2 receptors and H1 receptor

Compound	5-HT_{2A}R (nM)	5-HT_{2B}R (nM)	5-HT_{2C}R (nM)	H1 (nM)
**1**	> 10,000	501.0	2727.0	-
**2**	-	-	-	-
**3**	327.0	83.0	292.0	4.8
**4**	> 10,000	612.0	> 10,000	153.0
**5**	> 10,000	219.0	> 10,000	362.0
**6**	> 10,000	285.0	> 10,000	16.0
**7**	15.0	46.0	345.0	1.0
**8**	-	-	-	-
**9**	> 10,000	> 10,000	447.0	29.0
**10**	> 10,000	> 10,000	> 10,000	351.0
**11**	631.0	134.0	248.0	6.4
**12**	-	-	-	-
**13**	-	-	-	-
**14**	> 10,000	606.0	> 10,000	27.0
**15**	> 10,000	> 10,000	> 10,000	865.0
**16**	-	-	-	-
**17**	> 10,000	> 10,000	> 10,000	137.0
**18**	-	-	-	-
**19**	-	-	-	-
**20**	> 10,000	772.0	> 10,000	161.0
**21**	> 10,000	> 10,000	> 10,000	>10,000
**22**	623.0	306.0	4578.0	8.5
**23**	> 10,000	515.0	> 10,000	34.0
**24**	1,259.0	115.0	1,408.0	3.7
**25**	2,379.0	482.0	2,265.0	76.0
**26**	316.0	20.0	313.0	1.2
**27**	> 10,000	> 10,000	7,153.0	442.0
**28**	> 10,000	464.0	1084.0	44.0

Table S3: 5-HT_{2B}R binding compounds tested for antiseizure activity.

Compound	Main use	Drug Class	5-HT_{2A}R (nM)	5-HT_{2B}R (nM)	5-HT_{2C}R (nM)
**methylergonovine**	smooth muscle constrictor	serotonin	0.4	2.2	4.6
**BW-723C86**	Research use only	serotonin	89.7	3.2	114.8
**6-APB**	psychoactive drug	serotonin	1927.0	3.6	-
**Ro 60-0175**	Research use only	serotonin	37.2	4.3	9.1
**CP-809,101**	Research use only	serotonin	1.6	6.0	64.0
**norfenfluramine**	fenfluramine metabolite	serotonin	194.0	18.0	306.0
**mCPP**	psychoactive drug / trazodone metabolite	serotonin	54.5	30.3	13.0
**cabergoline**	hyperprolactinemia	dopamine	6.17	1.2	691.8
**bromocriptine**	Parkinson's disease	dopamine/ serotonin	107.1	56.2	741.3
**apomorphine**	Parkinson's disease	dopamine	120.2	131.8	102.3
**piribedil**	Parkinson's disease	dopamine	> 10,000	1,202.3	> 10,000
**TL-99**	Research use only	dopamine	2,344.2	2,041.7	2,290.9
